# Diagnostic evaluation and treatment of UTIs in children with neurogenic bladder

**DOI:** 10.1016/j.jpurol.2025.09.008

**Published:** 2025-09-15

**Authors:** Jacqueline G. Holden, Sowdhamini Wallace, Pearl W. Chang, Stephanie Davis-Rodriguez, Rana F. Hamdy, John M. Morrison, Michael J. Tchou, Victor Trevisanut, Vijaya Vemulakonda, Catherine S. Forster

**Affiliations:** a Division of Pediatric Hospital Medicine, Department of Pediatrics, University of Pittsburgh 4401 Penn Ave., Pittsburgh, PA, 15224, USA; b Division of Pediatric Hospital Medicine, Department of Pediatrics, Baylor College of Medicine and Texas Children’s Hospital, 6621 Fannin St, Houston, TX, 77030, USA; c Division of Pediatric Hospital Medicine, Department of Pediatrics, University of Washington, School of Medicine and Seattle Children’s Hospital, 4800 Sand Point Way NE, Seattle, WA, 98105, USA; d Division of Hospital Medicine, Department of Pediatrics, University of Cincinnati College of Medicine and Cincinnati Children’s Hospital, 3333 Burnett Ave, Cincinnati, OH, 45229, USA; e Division of Pediatric Infectious Diseases, Department of Pediatrics, Children’s National Hospital, 111 Michigan Ave NW, Washington, DC, 20010, USA; f Division of Pediatric Hospital Medicine, Department of Pediatrics, John Hopkins All Children’s Hospital, 501 6th Ave S, St. Petersburg, FL, USA; g Section of Hospital Medicine, Department of Pediatrics, University of Colorado School of Medicine and Children’s Hospital Colorado, 13123 East 16th St, Aurora, CO, 80045, USA; h Division of Urology, Department of Surgery, Oregon Health and Sciences University, Portland, OR, USA; i Division of Pediatric Urology, Department of Surgery, University of Colorado School of Medicine and Children’s Hospital Colorado, 13123 East 16th St, Aurora, CO, 80045, USA

**Keywords:** Urinary tract infection, Neurogenic bladder, Vesicoureteral reflux

## Abstract

**Objective:**

Children with neurogenic bladder (NGB) are at increased risk for urinary tract infections (UTIs), but there is a lack of guidelines to assist clinicians in diagnosing and treating these children. Our objective was to describe the presentation and treatment of provider diagnosed UTIs in children with NGB compared to children with vesicoureteral reflux (VUR) and to assess the proportion of children with NGB who met a consortium definition of UTI.

**Study design:**

We included children <18 years old with either VUR or NGB who were diagnosed in the emergency department with a febrile UTI in our multicenter retrospective cohort study. We extracted and compared UTI symptoms and urinalysis results specific to children with NGB to children with VUR. We measured the proportion of UTI diagnoses concordant with the Urologic Management to Preserve Initial Renal Function (UMPIRE) consensus definition of UTI, defined as ≥ 100,000 CFU/mL of 1 or 2 organisms, pyuria, and ≥ two symptoms of UTI.

**Results:**

The most common symptom among all children in the cohort was vomiting (38.8 %). Of the 215 children with NGB, 41.3 % met the UMPIRE definition for UTI. More children with NGB had multidrug resistant organisms (MDROs) cultured from their urine than those with VUR. Children with NGB, both who did and did not require CIC, had increased odds of MDRO in urine culture compared to those with VUR. Children with NGB were more likely to be prescribed broad-spectrum antibiotics than children with VUR.

**Conclusions:**

Most children with NGB diagnosed with febrile UTI in the ED did not meet a commonly recommended definition for UTI. The higher prevalence of MDRO UTIs and broad-spectrum antibiotic use in children with NGB highlights the need for accurate diagnostic approaches for UTI in this population, as well as the difficulty in diagnosing UTI in patients with NGB.

## Introduction

Urinary tract infections (UTIs) are one of the most common bacterial infections in children [1]. The diagnosis and management of UTIs in children without anatomic or functional anomalies of the urinary tract is standardized by national clinical practice guidelines [2,3]. Children with neurogenic bladder (NGB) are more prone to recurrent UTI; however, the diagnosis and management of UTI in this population is complicated as there are no widely-accepted clinical guidelines [4]. Diagnosis of UTI in children with NGB is challenging since this is a heterogeneous population with altered voiding dynamics. Additionally, symptoms that suggest a UTI in children with normal urinary function, such as urinary incontinence, can be normal in NGB while other symptoms, such as dysuria, may not be present in NGB due to alterations in sensation [4]. Finally, routine urinalysis (UA) has poor diagnostic accuracy in children with NGB, especially in those children who require clean intermittent catheterization (CIC) who may have chronic urothelial inflammation [5]. However, accurate UTI diagnosis is necessary to prevent secondary renal scarring and ultimately renal failure [6], while avoiding antibiotic overuse and the associated increased risk of multidrug resistant organisms (MDROs) [7-9]. Indeed, children with NGB have increased rates of resistant organisms in their urine, underscoring the need to improve diagnostic and antibiotic stewardship in these children [10,11].

Children with vesicoureteral reflux (VUR) share similar morbidities to those with NGB and are also at risk for renal scarring and MDROs in the setting of recurrent UTIs [12]. Little is known how diagnostic and treatment practices and rates of MDROs vary between children with NGB and children with high-grade VUR, as both populations are often excluded from studies. It is unknown whether these populations should have the same or unique diagnostic criteria and management protocols. Therefore, the objectives of this study were: 1) compare the symptomatology and laboratory findings between children with NGB and those with VUR and a provider-diagnosed UTI; 2) compare the prevalence of MDROs in urine culture in children with NGB and those with VUR; 3) to determine the proportion of children with NGB who meet a consensus definition of UTI [6]; and 4) describe differences in empiric treatment practices between children with NGB and those with VUR. We expected children with NGB would be less likely to meet the consensus UTI definition compared to children with VUR, and that children with NGB would have a higher prevalence of MDROs cultured from urine compared to those with VUR.

## Methods

This is a secondary analysis of data collected as part of a multicenter retrospective cohort study of children with anatomic and functional anomalies of the kidney and urinary tract that included six different children’s hospitals throughout the United States. Children who presented to the emergency department (ED) with an included anomaly (NGB or VUR) and were diagnosed with a UTI between 1/1/2017–12/31/2018 were included in the study. We initially identified children with both UTI and NGB or VUR based on *International Classification of Diseases, 10*th *revision, Clinical Modification* (ICD-10-CM) codes as previously described [13]. Eligibility was confirmed by chart review. We obtained institutional review board approval at all participating sites.

### Study population

The initial study included children <18 years of age who presented to the ED with either documented or reported fever (temperature ≥38 ° C) or hypothermia (<36 ° C) and were diagnosed with a UTI using a urine sample collected by clean catch, catheterization, or suprapubic aspiration. We included children with surgical management of their VUR. The initial exclusion criteria included children with meningitis, urine sample collected by bag, end-stage renal disease (defined as a child with a glomerular filtration rate less than 15, on active dialysis, or who had received a renal transplant), UTI in the last 30 days, genitourinary surgery in the past 7 days, or renal abscess. We also excluded children with foreign material that may have been in place from a recent genitourinary procedure that happened outside of the 7-day period. Additionally, children who were discharged to a subacute facility or transferred from another center were not included in the study. In this secondary analysis, we also excluded children who presented with hypothermia, although they were included in the initial study.

### Primary exposure groups

Our NGB population included patients with congenital (e.g., spina bifida) and acquired NGB (e.g., spinal cord injury (SCI)). We further divided this group into children who required CIC and those who did not require CIC. We compared children with NGB to children with isolated grade IV or V VUR. Children with both NGB and VUR were classified as NGB.

### Covariates

We collected data by manual extraction from medical charts, which were stored in a centralized REDCap database. The extracted data included patient demographics including age, sex, race, ethnicity, and insurance status. Patients were further characterized by catheterization regimen and use of UTI antibiotic prophylaxis.

### Diagnostic outcomes

We extracted the presence of common UTI symptoms, UA results, and urine culture results. Identified symptoms are listed in Table 2. We defined MDRO as organisms not susceptible to at least three antibiotic classes, including first and second generation cephalosporins, third and fourth generation cephalosporins, fluoroquinolones, sulfonamides, and aminoglycosides [13]. We described concordance of provider-diagnosed UTIs in our study population with the proposed definition from the Urologic Management to Preserve Initial Renal Function (UMPIRE), a protocol developed to offer best practice recommendations in the urologic care of people with spina bifida [6]. The UMPIRE definition requires ≥100,000 colony-forming units (CFU)/mL of 1 or 2 organisms, pyuria as evidenced by > 10 WBC on microscopy and/or ≥2+ LE on dipstick, and at least two UTI symptoms [6]. We also examined a modified UMPIRE definition with just laboratory parameters (e.g., pyuria and urine culture results) without symptoms given the lack of standardization around assessing and reporting symptoms in our retrospective design.

### Treatment outcomes

We examined antibiotic treatment covariates, including the antibiotic spectrum index (ASI), with a higher ASI indicating a broader-spectrum antibiotic [14], and duration of antibiotic treatment. We collected this data about all antibiotics, including empiric antibiotics started in the ED and any changes made by the inpatient team.

### Statistical analysis

In this study, we compared variables between children with NGB and those with VUR. We used descriptive statistics to characterize the cohort’s demographics, presenting symptoms, UA parameters, urine bacterial burden, and treatment. We used Student’s t-test to compare continuous variables between groups and Chi-square (or Fisher’s Exact, as appropriate) to compare categorical variables. To determine differences within categorical variables with more than 2 groups, we used the Bonferroni correction. We used logistic regression to determine the association of NGB with presence of MRDOs on urine culture. All analysis was completed at the encounter, not patient, level given the descriptive nature of this study. We used R Studio (version 1.1.447) for analysis.

## Results

### Sample characteristics

312 encounters met inclusion criteria for this study, 97 from children with VUR, 169 from children with NGB on CIC, and 46 from children with NGB not on CIC. Spina bifida was the most common etiology of NGB (n = 123, 57 %). There were differences in age, sex, and presence of cutaneous channel between groups (Table 1).

### Symptoms

The three most common symptoms in all groups of children, besides fever, was vomiting (39.2 % of those with VUR, 34.3 % of those on CIC, 54.3 % of those not on CIC), abdominal pain (19.6 % in VUR, 21.9 % in NGB on CIC and 30.4 % in NGB not on CIC), and fussiness (35.1 % in VUR, 17.8 % in NGB on CIC and 24.9 % in NGB not on CIC). There were children in all groups who had no symptoms other than fever (17.5 % in VUR, 17.2 % in NGB on CIC, and 19.6 % in NGB not on CIC) (Table 2).

### Urinalysis

There was no difference in categories of either LE or urine WBCs between groups, although 8.3 % of all children had no LE in their urine and 11.9 % had less than 5 urinary WBCs. Almost half (52.1 %) of children with NGB on CIC had positive nitrites on UA compared to 41.2 % of children with NGB not on CIC and 35.1 % of children with VUR (p = 0.02) (Table 2). UA parameters differed slightly between children who presented with and without vomiting within each group. Interestingly, children with VUR and NGB not on CIC who presented with vomiting tended had more inflammation on their UA compared to those without vomiting, though this trend was not seen in children with NGB on CIC. However, we did not use statistical testing given the low number of patients in this subanalysis and the fact that this was not a primary objective of the study (Supplemental Table 2).

### Urine culture

There was no difference in bacterial burden between groups (Table 3). MDROs were detected in a greater proportion in urine culture from children with NGB compared to those with VUR (6.2 % of children with VUR vs 17.1 % of children with NGB on CIC and 17.4 % of children with NGB not on CIC). When stratified by age, there was nearly a three-fold increase in the proportion of MRDOs in children with NGB on CIC compared to children with NGB not on CIC in children 7–17 years of age. Children with NGB, both those on CIC and not on CIC, had increased odds of MDROs compared to children with VUR, after controlling for age and sex (3.44 (1.29–10.40) NGB on CIC and 3.30 (1.05–10.87) for NGB not on CIC vs VUR) (Supplemental Table 1). We also looked at the proportion MDROs within samples with and without significant pyuria. MDROs were present in 15.5 % of samples with ≥50 urinary WBCs compared to 12.5 % of samples with <50 urinary white blood cells. Further, MDROs were also present in 11.3 % of samples with ≤10 WBCs.

Notably, there were no MDROs in children with VUR in children over the age of 7, and no extended-spectrum betalactamase (ESBL) bacteria cultured from urine from any children with VUR (Table 3). The only bacteria cultured from urine that were significantly different between groups were *Citrobacter* spp (8.7 % of cultures from children with NGB not on CIC versus 0 % of cultures from children with VUR versus 2.4 % of cultures from children with NGB on CIC, p < 0.01) and *Proteus* spp (15.2 % of children with NGB not on CIC vs 4.1 % of children with VUR and 3.6 % of children with NGB on CIC) (Supplemental Table 3).

In total, 65 children with NGB on CIC (38.5 %) and 24 (52.2 %) children with NGB not on CIC with provider-diagnosed UTI met the UMPIRE definition for UTI. 83 (49.1 %) children with NGB on CIC and 28 (60.9 %) children with NGB not on CIC met the modified definition. We did not apply either definition to children with VUR as neither definition is applicable to that population.

### Management

There was no difference in the mean duration of either intravenous (IV) or total antibiotic duration between groups in children admitted for management. More children with VUR were prescribed antibiotics with an ASI of 1–3 compared to children with NGB who did and did not require CIC (14.3 % vs 2.2 % vs 0 %, p < 0.01). More children with NGB on CIC were prescribed broader-spectrum antibiotics, with an ASI of ≥6 compared to children with NGB not on CIC or those with VUR (44.0 % vs 33.3 % and 11.9 % for ASI≥6, p < 0.01) (Table 4).

## Discussion

Here, we describe the variation in the diagnosis and treatment of UTI in children with NGB compared to children with VUR. We report that a minority of children with NGB on CIC diagnosed with a UTI during their encounter met the UMPIRE definition for UTI, while just over half of children with NGB not on CIC met the UMPIRE definition. In addition to noting variability in diagnostic and UA parameters, we also report that children with NGB had more MDROs and were treated with broader antibiotics compared to children with VUR. Our findings highlight the need to tailor diagnostic tools to more accurately diagnose UTI in children with NGB as improved diagnosis could reduce antibiotic overuse and resistance in this population.

While only a minority of children with NGB on CIC in our cohort met the full UMPIRE definition of UTI, a higher proportion met our modified definition based on pyuria and culture results. One possibility for the lower number meeting full criteria may be due to incomplete recording of symptoms. As this is a retrospective study, we were only able to rely on the symptoms reported in the medical record. Therefore, some symptoms may be underreported as clinicians may not have either asked or documented information about all of the symptoms included in the definition. Indeed, many of the symptoms seen among children in our study, including fever, abdominal pain, and back pain, are among the most common symptoms to be included in literature definitions of UTI [4]. Therefore, the presence of these symptoms alone may have alerted the clinician to the possibility of a UTI. Notably, the most common symptom documented within our cohort was vomiting, which is not referenced in the UMPIRE definition [6]. Among twenty-six articles evaluating UTI in children with spina bifida, only 3.8 % included vomiting in their definition of diagnosing UTI [4]. Despite few literature references to vomiting, it can be common in young children, with one study finding children under five who presented with vomiting had 1.6–1.7 times increased likelihood of UTI [15]. Given the prevalence of vomiting in our cohort, the presence of vomiting should be considered as a possible symptom of UTI in febrile children with NGB.

We determined that 62.7 % of children with NGB on CIC and 76.7 % of those with NGB not on CIC in our cohort had a positive urine culture with ≥100,000 CFU/mL of a known uropathogen. The American Academy of Pediatrics defines UTI in children as a positive urine culture of ≥50,000 CFU/ mL in the presence of pyuria, but this definition does not include children with NGB [8]. Although definitions used in the literature for children with NGB have moved to include the presence of symptoms and push the cutoff to ≥100,000 CFU/mL, there is new data supporting the reduction of the cutoff for a positive urine culture to be ≥ 10,000 CFU/mL for children with normal genitourinary anatomy [4,16]. It may be that ≥100,000 CFU/mL is more accurate for children with NGB, who require greater specificity in diagnosis, while children without NGB require increased sensitivity. However, future work is necessary to establish optimal urine culture criteria for UTI in children with NGB, as well as other parameters. For instance, urinary biomarkers, such as NGAL, have been shown to aid in the differentiation of UTI versus ASB [17].

The presence of pyuria alone is not sufficient for determining UTI, rather the absence of pyuria is a useful tool for ruling out UTI. In a longitudinal study of children with spina bifida who had ASB, 58 % of urine samples had >10 WBC in urine. Additionally, there was high variability of pyuria in individuals [18]. Other variables, such as presence of a cutaneous channel or associated VUR, have been shown to be associated with increased pyuria [19], which could confound UTI diagnosis. Furthermore, the Infectious Diseases Society of America remarks in their 2019 guideline that ASB can be associated with varying levels of pyuria and recommends using other criteria to distinguish between ASB and UTI in patients with NGB following SCI [20]. While 80.1 % of children with NGB in our study had >10 WBC, the presence of a positive urine culture and symptoms of UTI are additionally necessary for diagnosing UTI.

Upon appropriate diagnosis of UTI, proper management and treatment is critical in preventing antibiotic misuse. In this study, we used ASI to compare antibiotics between groups. The ASI is a useful metric for selecting appropriate antibiotics [14]. Broad-spectrum antibiotics (ASI≥6) were prescribed in 22.8 % of children with NGB, approximately twice the rate of their use in children with VUR. Additionally, in our cohort, children with NGB had a higher prevalence of MDROs and ESBL-producing organisms compared to children with VUR. It is difficult to fully assess the reason behind the higher rate of broad-spectrum antibiotic use in children with NGB within the context of this study, given the lack of full clinical data. It could be that children with NGB are more likely to have a history of MDROs, thus prompting clinicians to prescribe broader empiric antibiotics. As we report a difference in the rate of MDROs between children with NGB and with VUR, this is certainly possible. Further, many of those organisms do not necessitate an antibiotic with such a broad ASI. Thus, our data suggest that children with NGB may be given unnecessarily broad empiric antibiotics when compared to children with VUR, representing an opportunity to improve antibiotic stewardship in this population.

These findings have limitations that are due to the retrospective design of the study. The data pulled from medical records were limited by provider documentation and identification was limited on matching the appropriate ICD-10-CM codes. Children with NGB have unique symptoms of UTI, which could have been missed by clinicians looking for typical UTI symptoms, leading to the underestimation of children with at least two symptoms of UTI. We also did not collect additional clinical information, such as viral panels for upper respiratory infections, which are commonly tested for with UTI in children with NGB. Additionally, as all included sites were tertiary or quaternary care children’s hospitals, results may not be generalizable to other settings. We included children who had previous surgical management of their VUR, who may have differing presentation of UTI than children without surgical management. Another limitation is that the UMPIRE definition is specific for children <5-year-old with spina bifida, not all children with NGB. However, this restriction is likely related to the initial design of UMPIRE, which is to prospectively follow a birth cohort of children with spina bifida. It may not indicate the definition is inappropriate for children outside this age range or with acquired NGB. Finally, as children with NGB often have a higher rate of ED utilization, it is possible that children with NGB were oversampled in our cohort.

## Conclusion

We identified that children with NGB are prescribed broader spectrum antibiotics and have a higher prevalence of MDRO cultured from urine compared to children with VUR. Further, we highlight the difficulty in diagnosing UTI in children with NGB. A clinically accepted definition of UTI in children with NGB is necessary for appropriate diagnosis and treatment of UTI and will be critical for bladder management and renal preservation in patients with NGB. Further studies should evaluate whether vomiting should be incorporated as part of the definition of UTI for children with NGB.

## Supplementary Material

supplemental table 3

supplemental table 2

supplemental table 1

## Figures and Tables

**Table 1:** Comparison of characteristics of children with and without neurogenic bladder and physician diagnosis of urinary tract infection.

	Total Population (n = 312)	VUR (n = 97)	NGB with CIC (n 169)	NGB without CIC (n = 46)	p-value
Mean age (SD)	6.5 (5.6)	2.1 (3.2)	9.2 (4.9)	5.7 (5.9)	<0.01
1- <3 months, n (%)	21 (6.7)	17 (17.5)	1 (0.6)	3 (6.5)	<0.01
3 months-<2 years, n (%)	81 (26.0)	50 (51.5)	14 (8.3)	17 (37.0)	<0.01
2 – <7 years, n (%)	67 (21.5)	22 (22.7)	35 (20.7)	10 (21.7)	0.93
7–17 years, n (%)	143 (45.8)	8 (8.2)	119 (70.4)	16 (34.8)	<0.01
Sex					
Female, n (%)	191 (61.2)	68 (70.1)	91 (53.8)	32 (69.6)	0.02
Race					
White (n (%))	196 (62.8)	63 (65.0)	103 (60.9)	30 (65.2)	0.75
Black (n (%))	30 (9.6)	8 (8.2)	17 (10.1)	5 (10.9)	0.86
Asian (n (%))	9 (2.9)	3 (3.1)	6 (3.6)	0 (0.0)	0.44
Native American	2 (0.6)	1 (1.0)	1 (0.6)	0 (0.0)	0.76
Other/Not given	75 (24.0)	22 (22.7)	42 (24.9)	11 (23.9)	0.81
Ethnicity					
Hispanic (n (%))	102 (32.7)	31 (32.0)	56 (33.1)	15 (32.6)	0.81
Cutaneous channel^a^	45 (14.4)	0 (0.0)	42 (24.9)	3 (6.5)	<0.01
Antibiotic prophylaxis	92 (29.5)	37 (38.1)	48 (28.4)	7 (15.2)	0.06

NGB = neurogenic bladder; SD = standard deviation; VUR = vesicoureteral reflux; CIC = clean intermittent catheterization.

a Cutaneous channel refers to any surgically-created passage from the bladder to the skin, regardless of whether the children catheterizes through that channel.

**Table 2:** Diagnostic characteristics of children with and without neurogenic bladder and physician diagnosis of urinary tract infection.

	VUR (n = 97)	NGB with CIC (n = 169)	NGB without CIC (n = 46)	P-value
**Symptoms**				
Vomiting	38 (39.2)	58 (34.3)	25 (54.3)	0.04
Abdominal pain	19 (19.6)	37 (21.9)	14 (30.4)	0.25
Back pain	4 (4.1)	20 (11.8)	4 (8.7)	0.30
Dysuria	15 (15.5)	11 (6.5)	5 (10.9)	0.13
Urinary frequency	4 (4.1)	3 (1.8)	3 (6.5)	0.39
Pain with catheterization	–	5 (3.0)	1 (2.2)	0.77
Hematuria	2 (2.1)	12 (7.1)	0 (0.0)	0.15
Fussiness	34 (35.1)	30 (17.8)	11 (24.9)	0.95
Bladder Spasms	0 (0.0)	7 (4.1)	2 (4.3)	0.01
No symptoms other than fever	17 (17.5)	29 (17.2)	9 (19.6)	0.93
**UA parameters**				
LE none	8 (8.2)	11 (6.5)	7 (15.2)	0.17
LE trace	7 (7.2)	15 (8.9)	4 (8.7)	0.97
LE small	15 (15.5)	27 (16.0)	3 (6.5)	0.22
LE moderate	17 (17.5)	43 (25.4)	13 (28.2)	0.42
LE large	37 (38.1)	65 (38.5)	17 (37.0)	0.80
Positive nitrites	34 (35.1)	88 (52.1)	19 (41.3)	0.02
0-5 WBCs^a^	12 (14.6)	19 (13.0)	6 (16.2)	0.85
6-10 WBCs	6 (7.3)	17 (11.6)	3 (8.1)	0.55
11-20 WBCs	10 (12.2)	10 (6.8)	7 (18.9)	0.07
21-30 WBCs	11 (13.4)	29 (19.7)	6 (16.2)	0.47
31-50 WBCs	22 (26.8)	43 (29.3)	7 (18.9)	0.45
>50 WBCs	21 (25.6)	29 (19.7)	8 (21.6)	0.47
Composite negative UA^b^	4 (4.1)	4 (2.3)	3 (6.5)	0.35

We extracted the symptoms above from those documented in the chart. UMPIRE defines UTI symptoms in children with spina bifida as two or more of the following: fever, gross hematuria, abdominal, suprapubic or flank pain, new or worsening incontinence, new or worsening urinary urgency or frequency, pain with catheterization or urination, and malodorous or cloudy urine.

a 15 children with VUR, 22 children with NGB on CIC, and 9 children with NGB not on CIC did not have a microscopic UA sent.

b Negative UA was considered a UA with no LE, negative nitrites, and less than 5 white blood cells.

**Table 3:** A: Urine bacterial burden children with and without neurogenic bladder and physician diagnosis of urinary tract infection.

	VUR (n = 97)	NGB with CIC (n = 169)	NGB without CIC (n = 46)	p-value
**Bacterial burden**				
No growth	4 (4.1)	13 (7.7)	1 (2.2)	0.26
<50,000	21 (21.6)	21 (12.4)	5 (10.9)	0.11
50,000–100,000	13 (13.4)	29 (17.2)	5 (10.9)	0.49
>100,000	59 (60.8)	106 (62.7)	35 (76.7)	0.18
B: MDR and ESBL bacteria in children with and without neurogenic bladder and physician diagnosis of urinary tract infection.				
	VUR (n = 97)	NGB with CIC (n = 169)	NGB without CIC (n = 46)	p-value
**MDR bacteria**				
All ages	6 (6.2)	29 (17.1)	8 (17.4)	0.03
<3 months (n = 21)	1	0	1	
3 months-<2 years (n = 77)	4	3	5	
2 – <7 years (n = 62)	1	4	1	
7–17 years (n = 134)	0	22	1	
**ESBL bacteria** ^a^				
All ages (n = 132)	0 (0)	7 (5.3)	0 (0)	0.48

a ESBL status only available for 132 encounters.

**Table 4:** Inpatient treatment of children with and without neurogenic bladder and physician diagnosis of urinary tract infection.

	VUR (n = 42)	NGB on CIC (n = 91)	NGB not on CIC (n = 27)	P-value
No IV antibiotics	0 (0.0)	9 (10.0)	1 (3.7)	0.08
ASI for inpatient IV antibiotics				
1-3	6 (14.3)	2 (2.2)	0 (0.0)	<0.01
4-5	31 (73.8)	40 (44.0)	17 (63.0)	<0.01
≥6	5 (11.9)	40 (44.0)	9 (33.3)	<0.01
Mean duration of IV antibiotics (days)	3.6 (2.6)	3.8 (2.8)	3.9 (2.4)	0.92
Mean total duration of antibiotics (days)	11.7 (2.8)	10.6 (4.1)	11.2 (4.2)	0.47

152 children (55 with VUR, 78 with NGB on CIC, and 19 with NGB not on CIC) were discharged home from ED and excluded from this analysis.

## Appendix A. Supplementary data

Supplementary data to this article can be found online at https://doi.org/10.1016/j.jpurol.2025.09.008.
