# Supplementary material for: Diagnostic evaluation and treatment of UTIs in children with neurogenic bladder
Source: J Pediatr Urol. Author manuscript; Available in PMC 2026 Mar 17. (PMC12993934; doi:10.1016/j.jpurol.2025.09.008)
Supplement: supplemental table 3 [file NIHMS2144514-supplement-supplemental_table_3.docx]

Supplemental Table 3: Microbiology Results for Children with and without Neurogenic Bladder and Physician Diagnosis of Urinary Tract Infection

|  | VUR (n=97) | NGB on CIC (n=169) | NGB not on CIC (n=46) | p-value |
| --- | --- | --- | --- | --- |
| *Escherichia coli* | 46 (47.4) | 85 (50.3) | 24 (52.2) | 0.87 |
| *Klebsiella* spp | 19 (19.6) | 27 (16.0) | 5 (10.9) | 0.41 |
| Other | 10 (10.3) | 13 (7.7) | 4 (8.7) | 0.76 |
| *Proteus* spp | 4 (4.1) | 6 (3.6) | 7 (15.2) | <0.01 |
| No growth | 4 (4.1) | 12 (7.1) | 0 (0.0) | 0.13 |
| *Enterobacter* spp | 5 (5.2) | 9 (5.3) | 1 (2.2) | 0.66 |
| *Enterococcus* spp | 8 (8.2) | 8 (4.7) | 3 (6.5) | 0.51 |
| *Pseudomonas* spp | 1 (1.0) | 7 (4.1) | 3 (6.5) | 0.21 |
| *Citrobacter* spp | 0 (0.0) | 4 (2.4) | 4 (8.7) | <0.01 |
| *Staphylococcus* spp | 6 (6.2) | 5 (3.0) | 0 (0.0) | 0.13 |
| Mixed | 1 (1.0) | 5 (3.0) | 0 (0.0) | 0.32 |
| *Streptococcus* spp | 1 (1.0) | 1 (0.6) | 0 (0.0) | 0.76 |
| *Raoultella* spp | 0 (0.0) | 2 (1.2) | 0 (0.0) | 0.43 |
| Fungi | 0 (0.0) | 1 (0.6) | 0 (0.0) | 0.65 |
| *Aerococcus* spp | 1 (1.0) | 1 (0.6) | 0 (0.0) | 0.76 |
| *Morganella* spp | 1 (1.0) | 0 (0.0) | 0 (0.0) | 0.33 |
