# Supplementary material for: Diagnostic evaluation and treatment of UTIs in children with neurogenic bladder
Source: J Pediatr Urol. Author manuscript; Available in PMC 2026 Mar 17. (PMC12993934; doi:10.1016/j.jpurol.2025.09.008)
Supplement: supplemental table 2 [file NIHMS2144514-supplement-supplemental_table_2.docx]

Supplemental Table 2: Urinalysis Parameters in Children with Physician Diagnosis of Urinary Tract Infection who Present with and without Vomiting

|  | VUR | | NGB with CIC | | NGB without CIC | |
| --- | --- | --- | --- | --- | --- | --- |
|  | Vomiting (n=38) | No Vomiting (n=41) | Vomiting (n=58) | No Vomiting (n=85) | Vomiting (n=25) | No Vomiting (n=16) |
| LE none | 2 (5.3) | 5 (12.2) | 4 (6.9) | 6 (7.1) | 1 (4.0) | 5 (31.3) |
| LE trace | 2 (5.3) | 5 (12.2) | 2 (3.4) | 10 (11.8) | 3 (12.0) | 1 (6.3) |
| LE small | 4 (10.5) | 9 (22.0) | 11 (19.0) | 14 (16.5) | 1 (4.0) | 1 (6.3) |
| LE moderate | 7 (18.4) | 9 (22.0) | 17 (29.3) | 21 (24.7) | 7 (28.0) | 5 (31.3) |
| LE large | 19 (50.0) | 13 (31.7) | 24 (41.4) | 34 (40.0) | 11 (44.0) | 4 (25.0) |
| Positive Nitrites | 11 (29.0) | 20 (48.8) | 32 (55.2) | 52 (61.2) | 10 (40.0) | 9 (56.3) |
| <11 WBCs | 6 (19.4) | 10 (23.3) | 15 (28.8) | 17 (21.8) | 2 (10.0) | 5 (41.7) |
| 11-30 | 8 (25.8) | 13 (30.2) | 10 (19.2) | 23 (29.5) | 10 (50.0) | 2 (16.7) |
| 31-50 WBCs | 8 (25.8) | 12 (27.9) | 15 (28.8) | 22 (28.2) | 3 (15.0) | 3 (25.0) |
| >50 WBCs | 9 (29.0) | 8 (18.6) | 12 (23.1) | 16 (20.5) | 5 (25.0) | 2 (16.7) |
| Composite negative UA** | 1 (2.6) | 3 (7.3) | 3 (5.2) | 1 (1.2) | 0 (0.0) | 2 (12.5) |

Not all children had microscopic urinalysis completed. The following are the number of children in each category who had a microscopic UA completed: 31 for VUR andvomiting,43 for VUR and no vomiting , 52 for CIC with vomiting, 78 for CIC without vomiting, 20 for no CIC with vomiting, 12 no CIC no vomiting
