# Supplementary material for: Diagnostic evaluation and treatment of UTIs in children with neurogenic bladder
Source: J Pediatr Urol. Author manuscript; Available in PMC 2026 Mar 17. (PMC12993934; doi:10.1016/j.jpurol.2025.09.008)
Supplement: supplemental table 1 [file NIHMS2144514-supplement-supplemental_table_1.docx]

Supplemental table 1: Factors Associated with MDROs in urine culture

|  | Odds Ratio (95% confidence interval) |
| --- | --- |
| Age (years) | 0.99 (0.92-1.06) |
| Male | Ref |
| Female | 1.19 (0.60-2.42) |
| VUR | Ref |
| NGB on CIC | 3.44 (1.29-10.40) |
| NGB not on CIC | 3.30 (1.05-10.87) |
